# Magnetic resonance and ultrasound contrast imaging of polymer-shelled microbubbles loaded with iron oxide nanoparticles

**DOI:** 10.1098/rsos.160063

**Published:** 2016-08-03

**Authors:** Claudia Sciallero, Luca Balbi, Gaio Paradossi, Andrea Trucco

**Affiliations:** 1 Department of Electrical, Electronic, Telecommunications Engineering, and Naval Architecture, University of Genoa, Genoa, Italy; 2 Esaote S.p.A., Genoa, Italy; 3 Department of Chemistry, University of Rome Tor Vergata, Roma, Italy; 4 Pattern Analysis and Computer Vision, Istituto Italiano di Tecnologia, Genoa, Italy

**Keywords:** medical ultrasound, magnetic resonance imaging, dual-mode contrast agents, superparamagnetic iron oxide nanoparticles, polymeric microbubbles

## Abstract

Dual-mode contrast agents (CAs) have great potential for improving diagnostics. However, the effectiveness of CAs is strictly related to both the solution adopted to merge the two agents into a single probe unit, and the ratio between the two agents. In this study, two dual-mode CAs for simultaneous magnetic resonance imaging (MRI) and ultrasound imaging (UI) were assessed. For this purpose, different densities of superparamagnetic iron oxide nanoparticles (SPIONs) were anchored to the external surface of polymer-shelled microbubbles (MBs) or were physically entrapped into the shell. *In vitro* static and dynamic experiments were carried out with a limited concentration of modified MBs (10^6^ bubbles ml^−1^) by avoiding destruction during UI (performed at a peak pressure lower than 320 kPa) and by using a low-field MRI system (with a magnetic flux density equal to 0.25 T). Under these conditions, different imaging techniques, set-up parameters and SPION densities were used to achieve satisfactory detection of the CAs by using both UI and MRI. However, when the SPION density was increased, the MRI contrast improved, whereas the UI contrast worsened due to the reduced elasticity of the MB shell. For both UI and MRI, MBs with externally anchored SPIONs provided better performance than MBs with SPIONs entrapped into the shell. In particular, a SPION density of 29% with respect to the mass of the MBs was successfully tested.

## 1. Introduction

Among traditional medical imaging techniques, such as magnetic resonance imaging (MRI), ultrasound imaging (UI), computed tomography (CT) and radionuclide imaging (such as by positron emission tomography, PET), no single modality can be considered ideal, achieving all of the requirements for medical applications. Instead, different imaging modalities have complementary abilities in terms of sensitivity, resolution, penetration depth, throughput and complexity. As a consequence, significant improvement can be attained by using two or more techniques simultaneously. Such multi-modal imaging is a relatively recent idea that makes it possible to compensate for the limitations of single imaging modalities. The first fused PET/CT instrument was available commercially in 2001 [1], and the first commercial PET/MRI prototype was proposed in 2007 [2].

Imaging techniques often require specific contrast agents (CAs) to improve lesion and tissue characterization, anatomical visualization of body structures, morphological assessment, blood pool enhancement and perfusion imaging. Owing to these combined advantages, the development of dual-mode or multi-modal CAs is a useful way to obtain more valuable diagnostic information and to achieve more valuable treatment solutions [3]. In addition, multi-modal agents enable the administration of just one dose, instead of multiple doses, of different CAs, reducing potential side effects. Different strategies have been applied to achieve multi-modal functionality in a single probe unit. Multi-modality probe research is mostly based on nanomaterials, because their size and multi-component nature make it possible to combine probe materials for different imaging modalities [3]. In particular, nanoparticles for multi-modal imaging have been generated *ex novo* or have been generated based on existing nanoparticles to support simultaneous MRI/PET, PET/CT and CT/MRI [3].

In recent years, dual-mode agents for UI and MRI [4–9] that potentially combine the advantages of those two imaging techniques, or the high temporal resolution of UI and the high spatial resolution of MRI, have been proposed. For this type of CA, micro-devices and nanoparticles are used jointly: ultrasound CAs, composed of gas-filled microbubbles (MBs) protected by a shell [10], are complemented with nanoparticles (e.g. metal oxide, metal or semiconductor particles) that provide the desired magnetic response. Owing to their high stability and chemical versatility, polymer-shelled MBs [11–13] are usually preferred when designing dual-mode CAs. Alternative solutions based on polymeric nanocapsules with a liquid perfluorooctyl bromide core [3,14] or a perfluorocarbon nanoparticle emulsion [15] have also been proposed to develop MRI/UI dual-mode CAs, even if the solid, incompressible nanoparticles significantly reduce the ultrasound backscattering efficiency (i.e. the CAs' echogenicity) [16,17].

In dual-mode medical imaging, the requirements for the CA concentration can vary significantly between the two modalities [3,16]. Therefore, the enhancement of one modality could be at the expense of the other if the ratio between the two agent types or the solution adopted to merge the agents into a single probe is not properly controlled. Although contrast enhancement in MRI depends on the density of the magnetic nanoparticles and their level of aggregation, the MB echogenicity observed in UI is strictly related to the shell elasticity. In turn, the shell elasticity is affected by the addition of the magnetic nanoparticles. Both the strategy used to load the nanoparticles, and the nanoparticle density affect the MB shell characteristics and also affect the MRI/UI performance. A preliminary acoustic characterization of a dual-mode CA based on polymer-shelled MBs and a related UI assessment have experimentally confirmed that the strategy adopted to load the nanoparticles modifies the MB shell stiffness and, consequently, the ultrasound CA echogenicity [18,19].

In this paper, we focus on MRI/UI dual-mode CAs assembled by combining polymer-shelled MBs and superparamagnetic iron oxide nanoparticles (SPIONs) according to two recently proposed strategies [7]. SPIONs are materials used to provide negative contrast in clinical *T*_2_-weighted MRI; these are anchored to the external MB poly(vinyl alcohol) (PVA) surface by a coupling reaction or by entrapment into the shell during MB formation [7]. In this particular study, different nanoparticle densities were considered. The objective of our study was to detect the presence of CA by both MRI and UI, especially under difficult working conditions. These conditions involve low-field MRI (i.e. a magnetic flux density of 0.25 T) and a limited MB concentration (approx. 10^6^ MBs ml^−1^). Under these conditions, the nanoparticle loading strategy and the nanoparticle density that offered the best balance between MRI and UI contrast enhancement were identified through *in vitro* static and dynamic experiments. This investigation was performed simultaneously with optimization of the contrast imaging techniques and the related set-up parameters for both the MRI system and the UI system. The contrast imaging techniques that were considered for UI were specifically those that avoid MB destruction by subjecting the CA to limited pressure oscillations (i.e. an acoustic peak pressure lower than or equal to 320 kPa, corresponding to a mechanical index (MI) ≤ 0.15).

This study is important because it identifies CA assembly set-ups and techniques for MRI and UI that enable the simultaneous use of low-field MRI systems and UI medical scanners to investigate body regions, where MBs cannot reach high concentrations. For instance, medical investigation of rheumatoid arthritis benefits from the complementary information provided by MRI and UI (i.e. detection of bony erosions and measurement of cartilage thickness by MRI and assessment of synovial proliferation by UI) [20,21]. In addition, ultrasound CAs make it possible to evaluate the vascularization of synovial proliferation, whereas magnetic resonance CAs are used to enhance the early diagnosis of this disease [22–24]. Thus, simultaneous investigation with dual-mode CAs is particularly attractive for rheumatoid arthritis assessment, provided that limited MB concentrations (an unavoidable condition when small vessels are considered) and low-field MRI systems (frequently preferred for the analysis of extremities) can be accepted [21,23].

The contribution of this paper is innovative because in contrast to studies in the literature that have examined polymer-shelled MBs loaded with magnetic nanoparticles [4–6,8,9], we adopted a low-field MRI system and consider the same MB concentration and nanoparticle density for both imaging modalities; this makes it possible to perform simultaneous dual-mode medical investigations. In table 1, the dual-mode CA characteristics and the set-up parameters used for *in vitro* testing in this study and in previous studies are summarized and compared. In one study, Yang *et al*. [4] developed two-layer polymer-shelled MBs with SPIONs entrapped into the shell and assessed UI at a peak pressure of 187 kPa combined with high-field MRI via *in vitro* experiments. A high MB concentration and nanoparticle density were adopted for the MRI/UI tests. In another study, Park *et al*. [5] compared the UI results obtained when different nanoparticle materials (including SPIONs, gold and silica) were used to externally coat polymer-shelled MBs. Unfortunately, the nanoparticle density, acoustic peak pressure and contrast imaging technique were not reported. For the MBs coated with SPIONS, certain high-field MRI tests were also performed by varying the nanoparticle density. Liu *et al*. [6] investigated polymer-shelled MBs with SPIONs entrapped into the shell at different densities, and this CA was studied before and after ultrasound-induced MB destruction. However, the MB concentration and SPION density used to carry out MRI experiments were different from those used to test UI, and information regarding the acoustic pressure used was not provided. Meanwhile, He *et al*. [8] modified a proposed CA [4] to compare the two nanoparticle loading options: SPIONs entrapment into the shell and anchoring to the shell. UI and high-field MRI tests were performed *in vitro* by using high MB concentrations and nanoparticle densities. Finally, Cheng *et al*. [9] introduced polymer-shelled nanodroplets filled with fluorescent dyes and SPIONs as a multi-modal CA designed for UI, MRI and fluorescence imaging. Ultrasound irradiation was used to transform the nanodroplets into nanobubbles and MBs with SPIONs entrapped into the shell, and UI was tested via *in vitro* and *in vivo* experiments. Additionally, an acoustic pressure lower than 300 kPa was used to compare the polymer-shelled nanodroplets and a commercially available ultrasound CA (i.e. SonoVue, Bracco Research SA, Geneva, Switzerland). MRI tests were also performed both *in vitro* and *in vivo* using a high-field imaging system and different SPION densities. However, the relevant nanodroplet concentrations are not available.

**Table 1.** Dual-mode CAs, set-up parameters and operating conditions for *in vitro* MRI and UI tests according to different studies. n.a., not available. *MBN-into*, MBs with nanoparticles entrapped into the shell; *MBN-on*, MBs with nanoparticles anchored to the shell; *END-into*, encapsulated nanodroplets filled with fluorescent dyes and SPIONs (transformed into nanobubbles and MBs by ultrasound irradiation); PLA, poly(dl-lactide); PVA, poly(vinyl alcohol); PBCA, poly(butyl cyanoacrylate); PLGA, poly(lactic-co-glycolic acid); PEG, poly(ethylene glycol).

paper	CA type	mean diameter (µm)	shell	UI: frequency and peak pressure	MRI: magnetic flux density	MB concentration (MBs/ml)	nanoparticle density (µg/ml)	notes
Yang *et al*. [4]	*MBN-into*	3.98	2 layers: PLA and PVA	3.5 MHz	7.0 T	7 × 10^8^	5.73–180.23	UI: standard B-mode imaging technique
				187 kPa				MRI: *T*_2_-weighted fast spin-echo.
Park *et al*. [5]	*MBN-on*	5	lysozyme-alginate mixture	n.a.	3.0 T	10^4^	UI: n.a.	UI: comparison of MBN-on loaded with different nanoparticles (SPIONs, gold, or silica)
				n.a.			MRI: 0.47–4.7^a^	MRI: *T*_2_*-weighted fast field-echo; comparison of MBs with different SPION densities
Liu *et al*. [6]	*MBN-into*	2.3	PBCA	25 MHz	3.0 T	UI: 4 × 10^4^	UI: 0.17–0.62 × 10^−3^^a^	UI: before and after ultrasound-induced destruction
				n.a.		MRI: approximately 10^9^	MRI: 20–80^a^	MRI: *T*_1_- and *T*_2_-weighted multi-spin-echo before and after ultrasound-induced destruction
He *et al*. [8]	*MBN-on*	n.a.	PVA	3.5 MHz	7.0 T	UI: 1.8 × 10^8^	91.73	UI: standard B-mode imaging technique
	*MBN-into*			n.a.		MRI: 0.18–1.8 × 10^8^		MRI: *T*_2_-weighted multi-slice multi-echo
Cheng *et al*. [9]	*END-into*	0.385	2 layers: PLGA-PEG-PLGA and PVA	3.5 MHz	3.0 T	n.a.	UI: n.a.	UI: comparison between proposed nanodroplets and commercial ultrasound CAs
				243 kPa			MRI: 2–20^a^	MRI: *T*_2_-weighted spin-echo
This paper	*MBN-on*	3.8	PVA	4.5 MHz	0.25 T	10^6^	2–5^b^	UI: comparison of contrast-enhanced techniques
	*MBN-into*			230–320 kPa				MRI: *T*_2_*-weighted gradient-echo

^a^Approximate values computed from set-up parameters.

^b^SPION percentage of 15–38% with respect to the mass of a single MB.

The current paper is organized as follows. First, the characteristics of the investigated CAs, the signal processing techniques used for MRI and UI, and the related experimental set-up are described. Then, the obtained results, a discussion of the results and the best options that were identified are presented. Finally, several conclusions are drawn.

## 2. Material and methods

### 2.1. Two versions of dual-mode contrast agents

MBs are the most popular CAs for ultrasound due to their gas core, which has excellent acoustic backscattering properties. Recently, in addition to traditional soft-shell MBs, such as lipid MBs, polymeric shell MBs have been proposed [11–13]. A shell composed of polymer is generally thicker and more robust, providing higher stability and preventing gas diffusion.

A recently developed air-filled polymeric MB based on PVA, which has demonstrated chemical versatility, echogenicity and no toxicity [13,25,26], has been used to create a dual-mode micro-device for MRI and UI [7]. In particular, SPIONs with an average particle size of 8–10 nm have been coupled to the polymeric shell of these MBs. Two different strategies for loading MBs with SPIONs have been proposed [7], and the related dual-mode CAs are considered here. First, the SPIONs are functionalized to permit covalent binding to the polymeric shell, and they are then attached to the shell surface. This dual-mode CA will be referred to as *MBN-on*. Second, the SPIONs are added to a telechelic PVA solution immediately before MB formation, resulting in physical entrapment into the shell, with a uniform distribution. These SPIONs are specifically embedded in the shell during the cross-linking reaction at the water/air interface. This dual-mode CA will be referred to as *MBN-into*.

#### 2.1.1. Materials

Ferric chloride hexahydrate (FeCl_3_·6H_2_O, purity > 99%), ferrous chloride tetrahydrate (FeCl_2_·4H_2_O, purity > 99%, (3-aminopropyl) trimethoxysilane (APTMS), sodium cyanoborohydride (NaBH_3_CN) and sodium metaperiodate (NaIO_4_) were from Sigma-Aldrich (Milan, Italy). Low-molecular weight chitosan (CHIT) with a Brookfield viscosity of 20 000 cP, and a number-average molecular weight of 50 000 ± 5000 g mol^−1^ and PVA with a number-average molecular weight of 30 000 ± 5000 g mol^−1^, as determined by membrane osmometry, and a weight-average molecular weight of 70 000 ± 10 000 g mol^−1^, as determined by static light scattering, were purchased from Sigma-Aldrich (Milan, Italy). The acetylation degree of CHIT was determined to be 15% (mol/mol repeating units) by proton nuclear magnetic resonance (^1^H NMR) spectroscopy at 300 MHz (Bruker Advance, Milan, Italy). The inorganic acids and bases used were reagent-grade products from Carlo Erba (Milan, Italy). The water was Milli-Q purity grade (18.2 MΩ cm) and was produced using a deionization apparatus (PureLab, Steroglass, Perugia, Italy).

#### 2.1.2. Fabrication of dual-mode contrast agents

Synthesis of SPIONs has been reported previously [7]. Briefly, 5 ml of an aqueous solution of 1 M FeCl_3_ · 6H_2_O, 0.5 M FeCl_2_ · 4H_2_O and 0.4 M HCl served as a source of iron. The magnetite particles were coprecipitated under vigorous mechanical stirring (2000 r.p.m.) by adding the iron-containing solution to 50 ml of 0.5 M NaOH. After heating the alkaline solution to 80°C, the reaction was carried out for 30 min under an N_2_ atmosphere to prevent oxidation. The particles were then collected by sedimentation with the help of a large magnetic stir bar, followed by washing with degassed water and ethanol and vacuum drying.

For the synthesis of MBs with SPIONs attached to the PVA shell (i.e. *MBN-on*), nanoparticles were modified via silanization to introduce amino groups, which were able to react with the aldehyde groups of the MBs via reductive amination. For silanization, 100 mg of SPIONs was washed once with methanol (20 ml) and was subsequently washed with a mixture of methanol and toluene (20 ml; 1 : 1 v/v), followed by toluene alone (20 ml). The SPIONs were then dispersed into toluene (20 ml), and 0.5 ml of APTMS (3 mM in a methanol/toluene (1 : 1 v/v) mixture) was added to the SPION suspension. The suspension was further refluxed at 110°C for 24 hours under N_2_ flow and vigorous stirring. The modified particles were then magnetically collected, washed with methanol three times and vacuum dried. Typically, the ratio of the weights of the MBs to the silanized SPIONs was 1 : 2 (w/w). After being sonicated for 90 min in an ultrasound bath (‘Ultrasound cleaner’, CP104, CEIA, Italy) at a concentration of 20 mg ml^−1^, the SPIONs were added to 10 mg of MBs. Next, reductive amination with NaBH_3_CN at pH 5.0 was used to couple the reactive aldehydes on the MB shell with the amino groups of the APTMS moiety. The suspension was gently shaken for 5 days and exchanged with Milli-Q water. CHIT oxidation was then carried out by dissolving the polymer in water at a concentration of 1% (w/v) at pH 5.5 and oxidizing the C2 and C3 carbons of the CHIT repeating unit for 1 day using NaIO_4_ (GlcN/NaIO_4_ feed molar ratio of 1 : 0.5, where GlcN indicates the glucosamine repeating unit in the CHIT chain). Following conjugation with the silanized SPIONs, the oxidized repeating units of CHIT were coupled to the hydroxyl groups of the PVA shells by mixing a dispersion of 5 mg of MBs with 13 ml of the CHIT.

Finally, for the synthesis of MBs with SPIONs entrapped into the PVA shell (i.e. *MBN-into*), SPIONs without silanization were physically embedded in the shell by exploiting the favourable interactions between iron oxide nanoparticles and PVA [7,27]. More specifically, the SPIONs were resuspended in water at a concentration of 5 mg ml^−1^ and sonicated for 90 min using an ultrasound bath (see above), after which the SPIONs (at 20 or 40 mg ml^−1^, see next paragraph) were added during PVA shell formation. After 2 hours of homogenization at 8000 r.p.m. using an Ultra-Turrax equipped with a Teflon-coated head, the MBs incorporating the magnetic nanoparticles were washed with distilled water in a separatory funnel.

The amount of SPIONs bound to (*MBN-on*) or embedded in (*MBN-into*) MBs was estimated by thermogravimetric analysis (TGA) combined with differential thermal analysis [7], performed through an integrated coupling system (STA 409, Netzsch, Milan, IT). Lyophilized samples (20–30 mg) were placed in the TGA furnace and heated from 20°C to 1000°C at a rate of 10°C min^−1^ under nitrogen flux [7].

#### 2.1.3. Characteristics of dual-mode contrast agents

**Figure 1.**
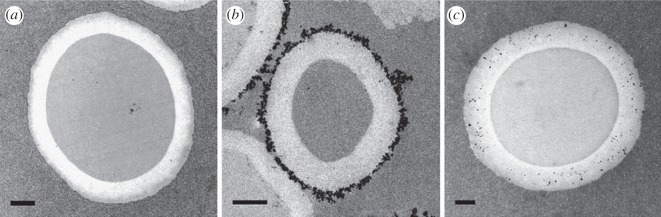
TEM images of the three types of CAs. From left to right: (*a*) cross-sections of *MB-plain*, (*b*) *MBN-on* (SPION weight percentage: 29%) and (*c*) *MBN-into* (SPION weight percentage: 15%) are displayed. The black dots represent SPIONs. Note that the cross-sections were obtained at random distances from the equatorial plane of the MBs, resulting in size differences between the analysed MBs. The scale bars represent 500 nm. Courtesy of IOS Press [27].

Confocal laser scanning microscopy was used to determine the bubble dimensions. No significant differences were observed between unloaded polymeric MBs (referred to as *MB-plain*), *MBN-on* and *MBN-into*, and an average diameter of 3.8 ± 0.6 µm was measured for both MB types. In figure 1, transmission electron microscopy (TEM) images showing cross-sections of the three types of MBs are shown; the black dots represent SPIONs. The SPIONs are clearly bound to the surface or embedded into the shell in the cases of *MBN-on* and *MBN-into*, respectively.

Because both the solution adopted to create dual-mode CAs, and the SPION density in each MB influence MRI and UI performance, magnetic MBs were prepared using different densities of nanoparticles. The considered percentages of SPIONs with respect to the weight of a single MB were 15 and 38% for *MBN-into* and 29% for *MBN-on.* and the related CAs will be referred to as *MBN-into15, MBN-into38* and *MBN-on29*, respectively. Table 2 summarizes the characteristics of the investigated CAs.

**Table 2.** Characteristics of the considered ultrasound CA and dual-mode CAs.

CA name	characteristics	application	SPIONs, % (w/w)
*MB-plain*	no SPIONs (unloaded)	UI	0
*MBN-into15*	SPIONs physically embedded into the shell	MRI and UI	15
*MBN-into38*	SPIONs physically embedded into the shell	MRI and UI	38
*MBN-on29*	SPIONs chemically linked to the shell	MRI and UI	29

### 2.2. Magnetic resonance imaging

MRI provides physiological and pathological information about living tissue, usually by exploiting the proton relaxation processes of water in biological systems [28]. Often, use of CAs is required to increase the sensitivity. The CAs used are divided into two classes: agents that decrease the *T*_1_ (longitudinal relaxation time) and therefore enhance the signal in *T*_1_-weighted images (positive contrast) and agents that reduce the *T*_2_ (transverse and spin–spin relaxation time) and therefore reduce the signal in *T*_2_-weighted images (negative contrast). The effectiveness of a CA is defined by the increase in the longitudinal and transverse relaxation rates (i.e. *R*_1_ = 1/*T*_1_ and *R*_2_ = 1/*T*_2_, respectively). The CAs for most commonly used MRI include paramagnetic complexes, paramagnetic ion nanoparticles (such as gadolinium- and manganese-based agents) and SPIONs [3]. In these cases, the contrast enhancement is due to magnetic field perturbation, which shortens the spin–spin relaxation time (*T*_2_), increasing the transverse relaxation rate (*R*_2_).

#### 2.2.1. Imaging techniques and relaxometric measurements

A gradient-echo sequence [28] (i.e. a pulse sequence with a high sensitivity to the overall transverse relaxation rate *R*_2_*, with *R*_2_* = *R*_2_ + *R*_2_’, where *R*_2_ and *R*_2_’ are the irreversible and reversible transverse relaxation rates, respectively) was used to obtain images. The gradient-echo sequence was chosen because of its superior sensitivity to the relaxation processes that act over distances of several micrometres, which is on the scale of vessels, cells and CAs [29]. In the case of dynamic tests, a flow compensation technique was applied (first order of gradient moment nulling) in the selection direction to reduce the risk of a flow artefact [28].

For the relaxometric measurements, preliminary static tests showed that due to the small size of the sample (for set-up details, see next subsection), the signal was too low to provide an adequate signal-to-noise ratio (SNR) in images, also taking into account the poor statistics that can be derived from a small number of image pixels. However, increasing the voxel size would have led to inaccurate localization, and the relaxation effects could have been overestimated due to intra-voxel de-phasing. Furthermore, a very large voxel size would be unrealistic in real-world applications. Therefore, to optimize SNR measurements, a modified gradient-echo sequence was implemented. The main differences with respect to a standard gradient-echo sequence were as follows: (i) only the readout gradient was used, (ii) no phase-encoding gradient was used, and (iii) no slice selection gradient was applied. By using the modified gradient-echo sequence, the signal was collected not from a single slice, but rather from the entire sample. This approach resulted in SNR improvement, which could be estimated to be of the order of 7000 (calculated as the ratio of the sample volume to the image voxel volume).

According to the exponential decay of signal with echo time (*T*_E_) [28] observed when the flip angle and the repetition time are fixed

2.1 $$S=S_{0}e^{-T_{E}/T_{2}^{*}},$$

where *S* is the signal intensity, and *S*_0_ is the signal intensity at *T*_E_ = 0, which can be considered as a constant factor (it only depends on fixed scan parameters). Equation (2.1) can be rewritten as follows:

2.2 $$\ln S=\frac{-T_{E}}{T_{2}^{*}}+\ln S_{0}.$$

The spin–spin relaxation time of the dual-mode CA suspensions, or *T*_2_*, could be estimated to perform relaxometric measurements. Because no phase encoding was used, the repetitions of the measurement (normally used to spatially encode the signal, by varying the amplitude of the phase-encoding gradient at each repetition) were used to evaluate the echo amplitude 256 times. From the repeated measurements, the average value and standard deviation of the echo amplitude were computed. Uncertainty about the independent variable (*T*_E_) was negligible. A least-square linear regression-fitting of the logarithm of the echo amplitude versus the echo time was performed according to (2.2), resulting in the linear slope, directly related to the value *T*_2_*. By applying error propagation, the average and standard deviation values for the relaxation time were calculated. Confidence intervals at 95% confidence level were then computed as intervals centred on the average value and with a half amplitude equal to twice the standard deviation.

#### 2.2.2. Experimental set-up

To investigate the bubble concentration, the SPION density and the loading strategy enabling MRI detection of the dual-mode CAs, *in vitro* experiments were performed with a 0.25 T MRI system (S-scan, Esaote S.p.A., Genoa, Italy). The tests were performed under both static and dynamic conditions using an optimized version of the dual phased-array wrist coil (Esaote S.p.A.).

For the static imaging experiments, the same MB solutions as those used for ultrasound experiments were transferred to test vials with a 12 mm diameter. Each solution was carefully stirred to ensure sample uniformity, but the formation of air bubbles was avoided. The samples were then positioned inside the scanner (with the static magnetic field perpendicular to the vial's long axis). The longest *T*_E_ available for the sequence and a long repetition time (*T*_R_) were selected to yield strong *T*_2_*-weighted contrast in a reasonable scan time (i.e. slightly above 2 min). The selected parameters were *T*_R_ = 900 ms and *T*_E_ = 34 ms. The images were obtained with a matrix size of 256 × 192, a field of view of 200 × 200 mm and a slice thickness of 6 mm.

The dynamic imaging tests were performed by using a suitable phantom that was designed to present continuous flow to simulate the blood circulation in vessels. In particular, a flow channel with an 8 mm diameter was encapsulated in a soft rubber matrix to allow for both UI and MRI, and a proper phantom holder was designed and built to permit placement of the phantom into a dedicated receiving coil for wrist examinations. In this way, the pipe was perfectly centred in the coil. No brisk flow speed variations were present, preventing flow artefacts in the MRI analysis. As a consequence, the MB solutions were pumped through the flow phantom at a constant flow rate of 0.5 ml min^−1^ by means of a peristaltic pump. The piping had to be long enough to keep the peristaltic pump at a safe distance from the magnet. Otherwise, the same imaging parameters as those introduced in the static experiments were employed.

### 2.3. Ultrasound imaging

Shelled MBs are efficient ultrasound CAs because when excited by an ultrasound wave, they undergo shell oscillations, expanding and contracting in response to the acoustic pressure changes [10]. At typical values of peak pressure used in UI, the MB oscillation is nonlinear (i.e. compression and expansion are not symmetric), and it generates an echo signal that contains harmonics at the excitation frequency. The nonlinear response is generally exploited by contrast-specific UI techniques to differentiate MBs from biological tissues because at such pressure values, tissues act mainly as linear systems. The composition and structure of the MB shell determine the related mechanical properties and the effect on the acoustic response of the MB population. Because CAs for simultaneous MRI and UI are obtained by modifying the shells of MBs originally developed as ultrasound CAs, the effectiveness of these dual-mode CAs in UI needs to be verified.

#### 2.3.1. Contrast-specific ultrasound imaging techniques

The acoustic detection of polymeric MBs (with or without the inclusion of SPIONs) has been investigated by comparing different UI techniques [19,25]. An important finding was that conventional contrast-specific UI techniques that are based on the exploitation of the nonlinear component of the second order (e.g. the well-known pulse inversion technique [30]) are not well suited to detecting polymeric MBs at a low concentration. Under the same working conditions, better performance was obtained by using UI techniques that exploit the nonlinear component of the third order. In particular, the contrast pulse sequence (CPS) [31], based on the transmission of three pulses for each line of sight (referred to as CPS3), provided the best performance, especially when the transmitted pulses were chirp signals (in this case, the technique is referred to as Chirp CPS3).

In CPS3, three pulses are transmitted in temporal order and are scaled in amplitude by the coefficients 0.5, −1 and 0.5. Related echoes are summed, which abates the linear contributions and preserves the second- and third-order nonlinear components. Because the third harmonic generally falls outside the ultrasound probe bandwidth, it was demonstrated [31,32] that good results are achieved by exploiting the third-order nonlinear component centred around the excitation frequency, which is captured through a suitable band-pass filter. If a chirp is used as transmission pulse, a matched filter should be applied after the echo summation to fully exploit the *BT* product (i.e. the bandwidth-by-time duration product), which is greater than one [32]. If the impulse response of this filter is *a*^3^(*t*) · *c*(–*t*), where *a*(*t*) is the pulse envelope, and *c*(*t*) is the chirp waveform, the filter can make it possible to capture the third-order nonlinear component centred around the excitation frequency, to compress the chirp time duration, and to increase both the SNR and the contrast-to-tissue ratio (CTR).

#### 2.3.2. Experimental set-up

The *in vitro* UI experiments were carried out with a MyLab Twice medical ultrasound scanner (Esaote S.p.A.), which was connected to an LA332 linear array probe (Esaote S.p.A.) and equipped with a grabber board for research purposes. This board was expressly designed to collect radio frequency (RF) signals. To enable multi-pulse transmission with arbitrary waveforms, a modified version of the commercial MyLab Twice scanner was developed. Moreover, an optical data link was used to transfer the acquired RF signals to an external PC, where they were stored and processed off-line.

A general set-up similar to that used for the dynamic MRI experiments was adopted. A UI phantom (Model 524, ATS Laboratories Inc., Bridgeport, CT, USA) with a 6 mm diameter flow channel embedded at a depth of 1.7 cm was imaged by using the linear array probe (central frequency of 5.5 MHz and fractional bandwidth of 95%). A peristaltic pump was used to guide diluted MBs from a beaker through the flow channel at a constant flow rate of 0.5 ml s^−1^.

For the CPS3 technique, the transmitted waveform was obtained by modulating a 4.5 MHz carrier through a truncated Gaussian envelope that encompassed five sinusoid cycles. The resulting fractional bandwidth was 40%. For the Chirp CPS3 technique, the transmitted waveform was a linear chirp pulse with a duration of 10 µs, a central frequency of 4.5 MHz and a raised cosine envelope encompassing 45 sinusoid cycles. The resulting fractional bandwidth was 40%. For reception, a band-pass filter ranging from 3 to 6 MHz was used for CPS3, and a matched filter with the impulse response described above was used for Chirp CPS3.

The experiments were performed by producing two different values of negative peak pressure at a focus depth of 1.7 cm, or 230 and 320 kPa, for both CPS3 and Chirp CPS3. These values are referred to as the pulses transmitted with the unitary scaling coefficient.

The ultrasound images were generated by processing the acquired RF signals off-line by applying the processing scheme described above and by implementing the same functions as those used on the back end of the scanner system (i.e. envelope detection and logarithmic compression).

## 3. Results and discussion

### 3.1. Contrast agent concentration

The MB concentration in a given solution is a crucial parameter not only for imaging purposes but also for pharmacological and toxicological reasons. The dosage should be as low as possible to avoid collateral effects and, at the same time, sufficient to enable reliable CA detection. In the case of dual-mode CAs, the MB concentration and percentage of SPIONs with respect to the weight of a single MB should be chosen to guarantee acceptable performance for both imaging modalities.

Acoustic characterization of magnetic MBs demonstrated that the dependence of backscattered power (i.e. the echogenicity) on the MB concentration is complex [19]: when the concentration was increased, saturation of the backscattered power was observed for MBs with embedded SPIONs (*MBN-into*) starting from 2 × 10^6^ MBs ml^−1^, whereas a backscattered power decrease was observed for MBs coated with SPIONs (*MBN-on*) starting from 10^6^ MBs ml^−1^. Overall, concentrations ranging between 4 × 10^5^ and 4 × 10^6^ MBs ml^−1^ are recommended for optimized UI.

To understand whether this concentration interval is acceptable for low-field MRI as well, certain preliminary static MRI tests were performed. Because the *T*_2_* effect of the CA is expected to be independent of the static field strength, low-field MRI can be considered a worst-case scenario, especially in terms of the SNR. The experimental findings are expected to be extendable to higher field intensities. Relaxometric measurements were carried out on three different samples; specifically, *MBN-on29* was diluted to a concentration of 10^5^, 10^6^ and 10^7^ MBs ml^−1^. The suspensions were obtained by diluting the MBs, which were fabricated at a concentration of 1.80 × 10^8^ MBs ml^−1^, in de-ionized and degassed (Milli-Q) water. The lowest concentration (i.e. 10^5^ MBs ml^−1^) showed no visible effect on the MRI signal. In the other two cases (i.e. 10^6^ and 10^7^ MBs ml^−1^), significant contrast was obtained because a significantly shorter relaxation time was measured (when the concentration increased from 10^5^ to 10^6^ MBs ml^−1^, *T*_2_* decreased by approx. 10 ms). Therefore, an intermediate concentration was chosen, which also proved optimal for UI.

Based on these preliminary results, a concentration of 10^6^ MBs ml^−1^ was found to be suitable for both the UI and the MRI experiments described herein. At this MB concentration, the nanoparticle density ranged from approximately 2 to 5 µg ml^−1^, corresponding to a SPION content range of 15–38% (w/w) with respect to the dry shell.

### 3.2. *In vitro* magnetic resonance imaging experiments

**Figure 2.**
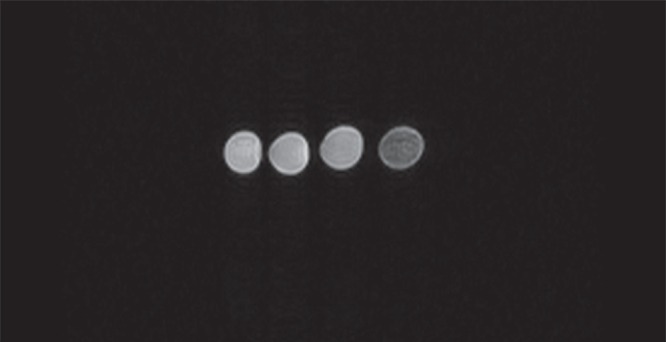
Low-field static MRI of the transverse section of four vials containing different CAs. From left to right: *MB-plain, MBN-into15, MBN-into38* and *MBN-on29*.

**Figure 3.**
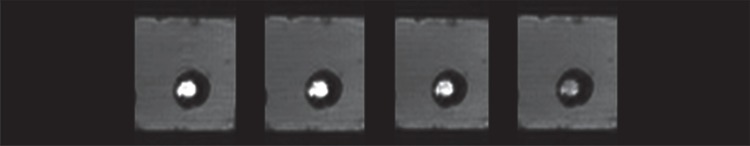
Low-field dynamic MRI of the phantom and four CAs. From left to right: *MB-plain, MBN-into15, MBN-into38* and *MBN-on29.*

The images shown in figure 2 were obtained from the static MRI experiments and were derived from vials containing the four different CAs listed in table 2. In figure 3, the dynamic MRI experiments performed using the phantom and the same CAs are shown. In *T*_2_*-weighted images, the contrast (namely, the negative contrast) increases when the image of the vial or the flow channel section becomes darker than when a solution containing MBs without magnetic nanoparticles is used.

The results reported in figures 2 and 3 for *MB-plain* are equivalent to those obtained for a saline solution: that is, no negative contrast was observed. The same figures show that MBs with embedded SPIONS (i.e. *MBN-into15* and *MBN-into38*) yielded only very limited negative contrast. By contrast, MBs coated with SPIONs (i.e. *MBN-on29*) yielded significant contrast, even if the iron content was lower than that in *MBN-into38*. In the static experiments (figure 2), the highly negative contrast of the SPION-coated MBs was more evident than in the dynamic experiments (figure 3) due to the absence of signal inhomogeneity generated by the flow.

**Figure 4.**
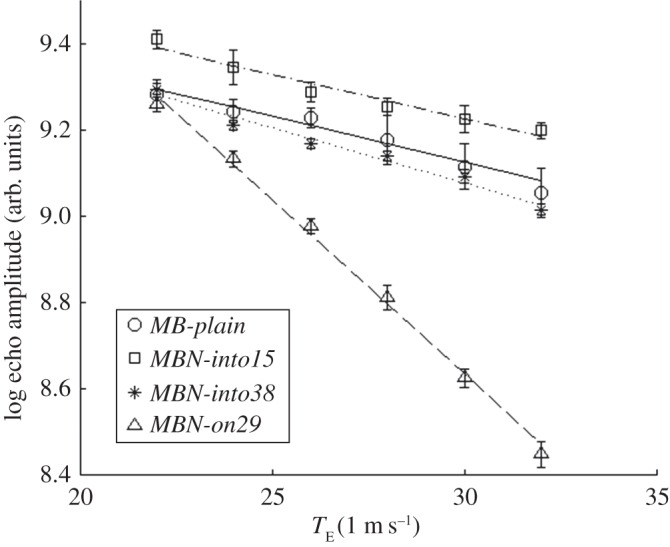
Logarithm of the echo amplitude (measured values and regression lines) in static T_2_*-weighted MRI as a function of echo time for the four CAs considered.

To quantify the different image contrast produced by the CAs, a static assessment of *T*_2_* relaxation parameters was performed. In particular, the four vials were used to measure the echo-time dependence of the gradient-echo signal, as previously described. The measured data are reported in figure 4, together with the regression line for each of the four CAs considered. From the slopes of the regression lines, the relaxation properties of the considered CAs can be easily derived. The related values are summarized in table 3. From the analysis of these relaxation properties, it is evident that *MBN-into15* did not significantly differ from *MB-plain* (because the paired *t*-test gives a *p*-value of 0.37), whereas the differences between *MBN-into38* and *MB-plain* were most likely to be statistically significant (*p*-value of 0.07). Meanwhile, the MBs coated with SPIONs, or *MBN-on29*, had the shortest *T*_2_* value, which was considerably lower than that of the other CAs, consequently, providing the strongest contrast.

**Table 3.** Relaxation properties of the different types of CAs.

CA type	SPIONs, % (w/w)	measured *T*_2_* (ms)	estimated *R*_2_* (s^−1^)
*MB-plain*	—	47 ± 11	21.2
*MBN-into15*	15	49 ± 6	20.4
*MBN-into38*	38	39 ± 2	25.6
*MBN-on29*	29	12.4 ± 0.4	80.6

### 3.3. *In vitro* ultrasound imaging experiments

**Figure 5.**
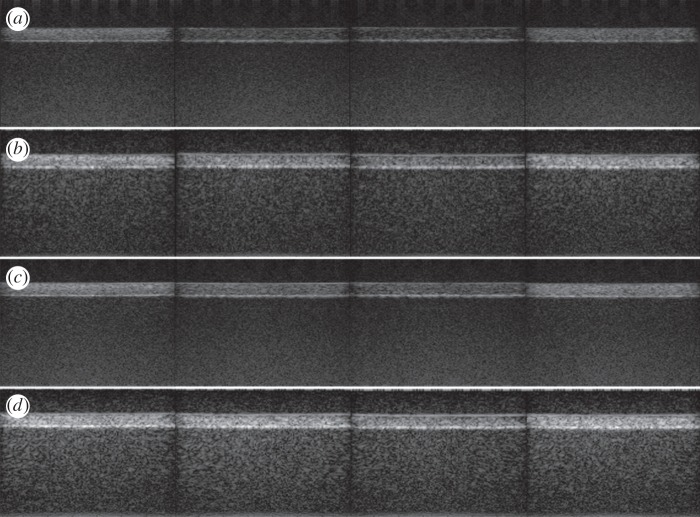
Ultrasound images of the phantom with the flow channel filled by different CAs, with different combinations of peak pressure and contrast-specific UI techniques. Peak pressure of: (*a*) 230 kPa and CPS3, (*b*) 230 kPa and Chirp CPS3, (*c*) 320 kPa and CPS3 and (*d*) 320 kPa and Chirp CPS3. From left to right: *MB-plain, MBN-into15, MBN-into38* and *MBN-on29.*

The same MB dilutions as those used to perform the MRI tests were employed in the UI experiments. The MBs of the four CAs listed in table 2 were pumped through the flow channel of the phantom and were imaged by using two contrast-specific UI techniques (i.e. CPS3 and Chirp CPS3) and two acoustic peak pressures (i.e. 230 and 320 kPa). In the ultrasound images obtained for the possible combinations, as shown in figure 5, different levels of contrast enhancement were observed. In UI, the contrast (namely, the positive contrast) is improved if the flow channel brightness increases with respect to that of the tissue-mimicking material, and the CTR can be used to quantify the achievement. The CTR is defined as the ratio between the energy of the signal received from a region containing a CA and the energy of the signal received from the surrounding tissue. We spatially filtered the processed RF signals by considering two regions of interest: inside the flow channel, for CAs, and above the flow channel, for tissue-mimicking material. In more detail, the acquired RF signals were converted to a two-dimensional matrix *M*(*i,j*), where *i* represents the *i*th line of sight of the image (*x*-axis), and *j* is the index of the time samples of each line of sight (*y*-axis). A rectangular region of interest (ROI) was defined in each image by considering *i* ranging between *i*_min_ and *i*_max_ (i.e. the desired lines of sight) and *j* ranging between *j*_min_ and *j*_max_ (i.e. the desired samples). Each signal segment between *j*_min_ and *j*_max_ was weighted by applying a Hamming window before further processing. A discrete Fourier transform of each signal segment was computed, and the mean energy spectral density of the ROI was calculated, as described in [19]*.* The two ROIs containing the bubbles and the tissue were defined by including 100 lines of sight (41 ≤* i *≤ 140, where the total number of lines of sight was 192) and two depth intervals (600 ≤ *j* ≤ 800 for tissue and 1050 ≤ *j* ≤ 1250 for bubbles, i.e. 201 time samples for each ROI, corresponding to a depth interval of 3 mm, immediately above the flow channel and inside the flow channel, respectively). The average energies from these regions were used to compute the CTR values reported in table 4.

**Table 4.** CTR values for the different types of MBs when the CPS3 and Chirp CPS3 techniques were used.

	CTR (dB)			
CA type	CPS3 230 kPa	CPS3 320 kPa	Chirp CPS3 230 kPa	Chirp CPS3 320 kPa
*MB-plain*	19.4	25.6	24.5	28.0
*MBN-into15*	12.2	18.5	22.3	27.7
*MBN-into38*	9.0	14.5	16.7	20.6
*MBN-on29*	19.1	25.9	26.6	28.7

All of the CTR values measured at 230 kPa were lower than the corresponding values measured at 320 kPa. In addition, for a given peak pressure value, the CTR values obtained by CPS3 were lower than the corresponding values obtained by Chirp CPS3.

Very similar performances were reported for *MB-plain* and *MBN-on29*: CTR differences lower than 2 dB were measured. The attachment of nanoparticles to the external surface of the MB shell did not significantly alter the UI performance, confirming previous findings [15]. The performance of *MBN-into* noticeably improved when the chirp pulse was adopted (a CTR improvement from 6 to 10 dB was observed) and decreased with the increase in SPION density. For *MBN-into15*, the CTR values were approximately 7 dB lower than those for *MB-plain* and *MBN-on29* when using CPS3, and they were from 1 to 4 dB lower when using Chirp CPS3. Instead, for *MBN-into38*, the CTR values were approximately 9 dB lower than those for *MB-plain* and *MBN-on29* when using CPS3, and they were approximately 8 dB lower when using Chirp CPS3. Overall, the acoustic performance of *MBN-into* was significantly lower than that of *MBN-on*, especially when conventional CPS3 was used.

Chirp CPS3 could be generally applied to improve the contrast in the images. It was particularly useful for achieving a satisfactory CTR for *MBN-into*. A drawback of using this coded technique is the weak loss of axial accuracy. For instance, the size of the flow channel, estimated from the ultrasound images, was 6.27 mm and 7.19 mm for CPS3 and Chirp CPS3, respectively, compared with the real diameter of 6 mm.

## 4. Discussion

*In vitro* MRI and UI experiments demonstrated that both the strategy used to load MBs with nanoparticles and the nanoparticle density affect the imaging performance.

Two SPION densities were used for MBs with nanoparticles embedded into the shell (i.e. *MBN-into*). The results obtained with the two imaging modalities showed that an increase in the SPION density increases the MRI contrast and decreases the contrast in UI. In particular, *MBN-into15* did not provide any contrast in MRI, and it provided UI performance that was significantly lower than that for unloaded MBs, i.e. *MB-plain*. However, the adoption of the chirp coding nearly eliminated the performance gap with respect to unloaded MBs. Meanwhile, *MBN-into38* provided only weak contrast in MRI and a severe contrast decrease in UI with respect to *MB-plain*. The adoption of the chirp improved the UI performance, but it only marginally reduced the performance gap with respect to unloaded MBs.

MBs externally coated with nanoparticles (i.e. *MBN-on*) were loaded with SPIONs at a density that was intermediate between the two densities used for *MBN-into*. In this case, satisfactory performance was obtained for both MRI and UI: the dual-mode CA *MBN-on29* provided UI contrast that was approximately equal to that provided by unloaded MBs, and it provided significant contrast in both static and dynamic *T*_2_*-weighted MRI.

The advantages of *MBN-on* with respect to *MBN-into* can be explained by analysing the related magnetic and mechanic characteristics. In MRI, the resonant spins (i.e. the hydrogen nuclei contained in water) can dynamically come into closer contact with SPIONs if the nanoparticles are placed on the shell surface, resulting in stronger and possibly more time-varying field inhomogeneity, which causes quicker magnetization de-phasing, i.e. a shorter *T*_2_*. In UI, the development of a dual-mode CA with SPIONs externally attached to the MBs does not significantly change the shell elasticity, whereas SPIONs entrapped into the shell produce stiffer MBs, negatively affecting the linear and nonlinear backscattering strength. This statement is supported by the measurements of the shear modulus and viscosity of the shell that were previously reported [19,33] for two dual-mode CAs that were conceptually analogous to *MBN-into* and *MBN-on*.

In summary, low-field MRI and low-pressure UI have been successfully applied to detect moderate concentrations of polymeric MBs externally coated with nanoparticles (i.e. *MBN-on29*). The longer blood circulation time for polymer-shelled CAs compared with commercially available lipid-shelled ultrasound CAs, such as SonoVue, offers more time for image acquisition [27], which enables multi-modal scanning. During a typical MRI examination, the post-contrast injection phase consists of a single scan, thus lasting 5–6 min. The enhancement is obtained through the comparison with the same scan acquired in the pre-contrast phase. This means that if a dual-mode examination should be performed, a UI session can be started immediately after the end of the MRI procedure, within the lifetime of the CAs. The CA shelf-life is increased thanks to the polymeric shell that offers both mechanical stability and chemical versatility. During UI *in vitro* experiments, we noticed that the MBs were still clearly detectable after more than 30 min. An analogous conclusion was reported in [11] where the echo intensity produced by polymeric MBs did not significantly decrease 20 min after injection. Finally, it is to be noted that the multi-modal approach is favoured by the use of a low-field MRI scanner, since all necessary equipment can be kept in close proximity, easing in this way the workflow.

## 5. Conclusion

*In vitro* experiments were performed to assess the performance of different dual-mode CAs, which were assembled by combining polymer-shelled MBs and SPIONs, when applied in simultaneous magnetic resonance and ultrasound contrast imaging. The SPIONs were anchored to the external MB surface by a chemical reaction (*MBN-on*) or were entrapped into the shell during MB formation (*MBN-into*). The specific objective was to detect the CAs when a limited MB concentration (i.e. approx. 10^6^ MBs ml^−1^) was used and when MRI was performed using a low-field system, and UI was performed to avoid MB destruction. Regarding the imaging techniques, a gradient-echo sequence was used to produce *T*_2_*-weighted magnetic resonance images, whereas the CPS3 scheme was used in UI to reveal and display the nonlinear component of the third order, possibly incorporating the transmission of chirp waveforms.

The CA *MBN-on29* provided significant contrast in both the static and the dynamic MRI tests, despite the limited MB concentration and SPION density. The ratio of the spin–spin relaxation time estimated for this CA to the relaxation time estimated for the same MBs without SPIONs was 4 (*MB-plain*). UI tests were performed at peak pressures of 230 and 320 kPa, with a CTR whose value was always greater than 19 dB and that was approximately equal to that observed with *MB-plain*. The CTR observed at 320 kPa was higher than that observed at 230 kPa. The difference was nearly eliminated when the chirp waveform was introduced, at the expense of limited degradation of the axial resolution.

By contrast, the CA *MBN-into* did not provide useful MRI performance, even when the SPION density was increased to 38%. Additionally, in UI, the observed CTR was noticeably lower than that observed with *MBN-on*, especially at a SPION density of 38%. Nevertheless, CTR values higher than 20 dB could be attained by adopting the chirp waveform and working at an incident pressure of 320 kPa.

## Data accessibility

Datasets [34] used in this research are available via the Dryad Repository (http://dx.doi.org/10.5061/dryad.8bp16).

## References

[1] Townsend DW, Beyer T, Blodgett TM 2003 PET/CT scanners: a hardware approach to image fusion. Semin. Nucl. Med. 33, 193–204. (doi:10.1053/snuc.2003.127314) 12931321 10.1053/snuc.2003.127314

[2] Cherry SR, Louie AY, Jacobs RE 2008 The integration of positron emission tomography with magnetic resonance imaging. Proc. IEEE 96, 416–438. (doi:10.1109/JPROC.2007.913502)

[3] Louie A 2010 Multimodality imaging probes: design and challenges. Chem. Rev. 110, 3146–3195. (doi:10.1021/cr9003538) 20225900 10.1021/cr9003538 PMC2878382

[4] Yang F, Li Y, Chen Z, Zhang Y, Wu J, Gu N 2009 Superparamagnetic iron oxide nanoparticle-embedded encapsulated microbubbles as dual contrast agents of magnetic resonance and ultrasound imaging. Biomaterials 30, 3882–3890. (doi:10.1016/j.biomaterials.2009.03.051) 19395082 10.1016/j.biomaterials.2009.03.051

[5] Park JI, Dinesh J, Ross W, Wendy O, Siyon C, Greg JS, Kumacheva E 2010 Microbubbles loaded with nanoparticles: a route to multiple imaging modalities. J. Am. Chem. Soc. Nano Lett. 4, 6579–6586. (doi:10.1021/nn102248g) 10.1021/nn102248g 20968309

[6] Liu Z, Lammers T, Ehling J, Fokong S, Bornemann J, Kiessling F, Gätjens J 2011 Iron oxide nanoparticle-containing microbubble composites as contrast agents for MR and ultrasound dual-modality imaging. Biomaterials 32, 6155–6163. (doi:10.1016/j.biomaterials.2011.05.019) 21632103 10.1016/j.biomaterials.2011.05.019

[7] Brismar TB et al. 2012 Magnetite nanoparticles can be coupled to microbubbles to support multimodal imaging. Biomacromolecules 13, 1390–1399. (doi:10.1021/bm300099f) 22458325 10.1021/bm300099f

[8] He W, Yang F, Wu Y, Wen S, Chen P, Zhang Y, Gu N 2012 Microbubbles with surface coated by superparamagnetic iron oxide nanoparticles. Mater. Lett. 68, 64–67. (doi:10.1016/j.matlet.2011.10.013)

[9] Cheng X, Li H, Chen Y, Luo B, Liu X, Liu W, Xu H, Yang X 2013 Ultrasound-triggered phase transition sensitive magnetic fluorescent nanodroplets as a multimodal imaging contrast agent in rat and mouse model. PLoS ONE 8, e85003 (doi:10.1371/journal.pone.0085003) 24391983 10.1371/journal.pone.0085003 PMC3877337

[10] Cosgrove D 2006 Ultrasound contrast agents: an overview. Eur. J. Radiol. 60, 324–330. (doi:10.1016/j.ejrad.2006.06.022) 16938418 10.1016/j.ejrad.2006.06.022

[11] El-Sherif DM, Wheatley MA 2003 Development of a novel method for synthesis of a polymeric ultrasound contrast agent. J. Biomed. Mater. Res. 66, 347–355. (doi:10.1002/jbm.a.10586) 10.1002/jbm.a.10586 12889005

[12] Lavisse S et al. 2005 *In Vitro* echogenicity characterization of poly[lactide-coglycolide] (PLGA) microparticles and preliminary *in vivo* ultrasound enhancement study for ultrasound contrast agent application. Invest. Radiol. 40, 536–544. (doi:10.1097/01.rli.0000170818.03210.ee) 16024992 10.1097/01.rli.0000170818.03210.ee

[13] Cavalieri F, Finelli I, Tortora M, Mozetic P, Chiessi E, Polizio F, Brismar TB, Paradossi G 2008 Polymeric microbubbles as diagnostic and therapeutic gas delivery device. Chem. Mater. 20, 3254–3258. (doi:10.1021/cm703702d)

[14] Pisani E, Tsapis N, Paris J, Nicolas V, Cattel L, Fattal E 2006 Polymeric nano/microcapsules of liquid perfluorocarbons for ultrasonic imaging: physical characterization*.* Langmuir 22, 4397–4402. (doi:10.1021/la0601455) 16618193 10.1021/la0601455

[15] Tran TD, Caruthers SD, Hughes M, Marsh JN, Cyrus T, Winter PM, Neubauer AM, Wickline SA, Lanza GM 2007 Clinical application of perfluorocarbon nanoparticles for molecular imaging and targeted therapeutics. Int. J. Nanomed. 2, 515–526. PMC2676820 18203420

[16] Hahn MA, Singh AK, Sharma P, Brown SC, Moudgil BM 2011 Nanoparticles as contrast agents for in-vivo bioimaging: current status and future perspectives. Anal. Bioanal. Chem. 399, 3–27. (doi:10.1007/s00216-010-4207-5) 20924568 10.1007/s00216-010-4207-5

[17] Parker KJ, Baggs RB, Lerner RM, Tuthill TA, Violante MR 1990 Ultrasound contrast for hepatic tumors using IDE particles. Invest. Radiol. 25, 1135–1139. (doi:10.1097/00004424-199010000-00013) 2079414 10.1097/00004424-199010000-00013

[18] Sciallero C, Trucco T 2013 Ultrasound assessment of polymer-shelled magnetic microbubbles used as dual contrast agents. J. Acoust. Soc. Am. 133, EL478–EL484. (doi:10.1121/1.4804942) 10.1121/1.4804942 23742443

[19] Sciallero C, Grishenkov D, Kothapalli S, Oddo L, Trucco A 2013 Acoustic characterization and contrast imaging of microbubbles encapsulated by polymeric shells coated or filled with magnetic nanoparticles. J. Acoust. Soc. Am. 134, 3918–3930. (doi:10.1121/1.4824337) 24180801 10.1121/1.4824337

[20] Backhaus M et al. 1999 Arthritis of the finger joints. Arthritis Rheum. 42, 1232–1245. (doi:10.1002/1529-0131(199906)42:6<1232::AID-ANR21>3.0.CO;2-3) 10366117 10.1002/1529-0131(199906)42:6<1232::AID-ANR21>3.0.CO;2-3

[21] Iagnocco A, Perella C, D'Agostino MA, Sabatini E, Valesini G, Conaghan PG 2011 Magnetic resonance and ultrasonography real-time fusion imaging of the hand and wrist in osteoarthritis and rheumatoid arthritis. Rheumatology 50, 1860–1864. (doi:10.1093/rheumatology/ker111) 21406468 10.1093/rheumatology/ker111

[22] Klauser A et al. 2005 Contrast enhanced gray-scale sonography in assessment of joint vascularity in rheumatoid arthritis: results from the IACUS study group. Eur. Radiol. 15, 2404–2410. (doi:10.1007/s00330-005-2884-9) 16132921 10.1007/s00330-005-2884-9

[23] Lindegaard H, Vallo J, Horslev-Petersen K, Junker P, Ostergaard M 2001 Low field dedicated magnetic resonance imaging in untreated rheumatoid arthritis of recent onset. Ann. Rheum. Dis. 60, 770–776. (doi:10.1136/ard.60.8.770) 11454641 10.1136/ard.60.8.770 PMC1753796

[24] Tan YK, Ostergaard M, Conaghan PG 2012 Imaging tools in rheumatoid arthritis: ultrasound vs magnetic resonance imaging. Rheumatology 51, vii36–vii42. (doi:10.1093/rheumatology/kes329) 23230093 10.1093/rheumatology/kes329

[25] Sciallero C, Paradossi G, Trucco A 2012 A preliminary *in vitro* assessment of polymer-shelled microbubbles in contrast-enhanced ultrasound imaging. Ultrasonics 52, 456–464. (doi:10.1016/j.ultras.2011.10.008) 22133737 10.1016/j.ultras.2011.10.008

[26] Cerroni B, Chiessi E, Margheritelli S, Oddo L, Paradossi G 2011 Polymer shelled microparticles for targeted doxorubicin delivery in cancer therapy. Biomacromolecules 12, 593–601. (doi:10.1021/bm101207k) 21235225 10.1021/bm101207k

[27] Härmark J, Larsson MK, Razuvajev A, Koeck PJB, Paradossi G, Brodin L-A, Caidahl K, Hebert H, Bjällmark A 2015 Investigation of the elimination process of a multimodal polymer-shelled contrast agent in rats using ultrasound and transmission electron microscopy. Biomed. Spectrosc. Imaging 4, 81–83. (doi:10.3233/BSI-140099)

[28] Haacke EM, Brown RW, Thompson MR, Venkatesan R 1999 Magnetic resonance imaging: physical principles and sequence design. New York, NY: Wiley.

[29] Kiselev VG 2005 Transverse relaxation effect of MRI contrast agents: a crucial issue for quantitative measurements of cerebral perfusion. J. Magn. Reson. Imaging 22, 693–695. (doi:10.1002/jmri.20452) 16261568 10.1002/jmri.20452

[30] Burns PN, Wilson SR, Simpson DH 2000 Pulse inversion imaging of liver blood flow: improved method for characterizing focal masses with microbubble contrast. Invest. Radiol. 35, 58–71. (doi:10.1097/00004424-200001000-00007) 10639037 10.1097/00004424-200001000-00007

[31] Phillips PJ 2001 Contrast pulse sequences (CPS): imaging nonlinear microbubbles. IEEE Ultrason. Symp. 2, 1739–1745. (doi:10.1109/ultsym.2001.992057)

[32] Crocco M, Pellegretti P, Sciallero C, Trucco A 2009 Combining multi-pulse excitation and chirp coding in contrast-enhanced ultrasound imaging. Meas. Sci. Technol. 20, 104017 (doi:10.1088/0957-0233/20/10/104017)

[33] Poehlmann M et al. 2014 On the interplay of shell structure with low- and high-frequency mechanics of multifunctional magnetic microbubbles. Soft Matter 10, 214–226. (doi:10.1039/C3SM51560E) 24651844 10.1039/c3sm51560e

[34] Sciallero C, Balbi L, Paradossi G, Trucco A 2016 Data from: magnetic resonance and ultrasound contrast imaging of polymer-shelled microbubbles loaded with iron oxide nanoparticles. Dryad Digital Repository. (doi:10.5061/dryad.8bp16) 10.1098/rsos.160063 PMC5108937 27853587

